# Supplementary material for: Spatiotemporal Evolution and Driving Forces of Vegetation Cover in the Urumqi River Basin
Source: Int J Environ Res Public Health. 2022 Nov 19;19(22):15323. doi: 10.3390/ijerph192215323 (PMC9690905; doi:10.3390/ijerph192215323)
Supplement: Supplementary file 1 [file ijerph-19-15323-s001.zip › ijerph-1949742-supplementary.pdf]

International Journal of
Environmental Research and Public Health

MDPI

Article

Spatiotemporal Evolution and Driving Forces of Vegetation Cover in the Urumqi River Basin

Supplementary Materials

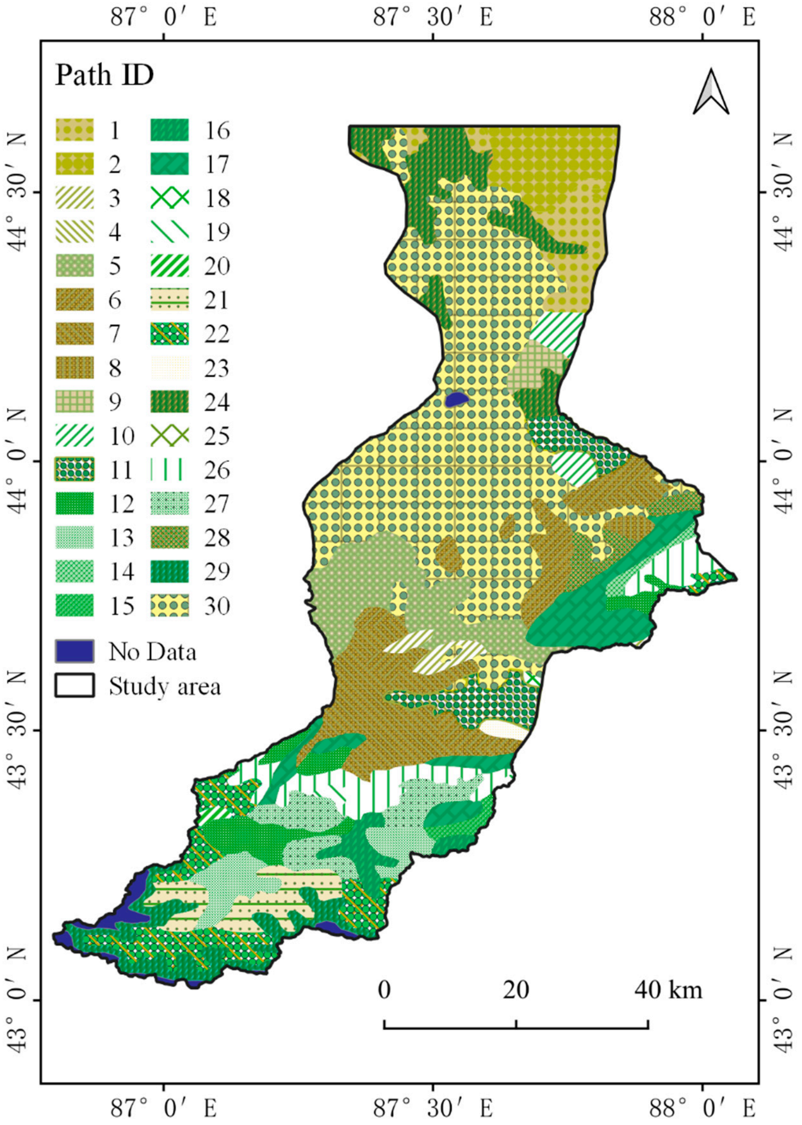

Figure S1. Distribution of vegetation types in the Urumqi River basin.

Table S1. Vegetation attribute table.

Family	Species	Path ID
Chenopodiaceae	*Haloxylon ammodendron*	1
	Haloxylon Persicum	2
	Ceratoides latens	3
	Camphorosma monspeliaca	11
	Anabasis brevifolia	4,11
	Nanophyton erinaceum	5
	Seriphidium transiliense	6
	Seriphidium borotalense	7,28
	Seriphidium santolinum	8
	Kalidium foliatum	9
	Suaeda dendroides	10
Gramineae	*Stipa capillata*	12,27
	Poa angustifolia	13,27
	Festuca valesiaca subsp	14
	Festuca ovina	15,28
	Agropyron cristatum	16
	Stipa caucasica	17
	Phragmites australis	18
	Triticum aestivum	30
	Zea mays	30
Rosaceae	*Rosa sericea Lindl*	19
	Cotoneaster adpressus	19
	Malus pumila Mill	30
Cyperaceae	*Carex songorica*	20
	Kobresia capillifolia	21
	Carex stenocarpa	22
Compositae	*Saussurea japonica*	29
	Cremanthodium reniforme	29
Pinaceae	*Picea schrenkiana*	26
Ephedraceae	*Ephedra przewalskii*	23
Tamaricaceae	*Reaumuria songarica*	24
Leguminosae	*Caragana stenophylla*	25
Crassulaceae	*Rhodiola rosea*	29
Cucurbitaceae	*Cucumis melo*	30
Vitaceae	*Vitis vinifera*	30

Table S2. Landsat images information.

year	Sensor	Image ID	Cloud Cover Percentage
2000	Landsat5_TM	LT05_142029_20000707	23
		LT05_142030_20000707	13
2001	Landsat5_TM	LT05_143029_20010523	0
		LT05_143029_20010523	1
2002	Landsat5_TM	LT05_142029_20020627	2
		LT05_143029_20020627	16
2003	Landsat5_TM	LT05_142030_20030529	3
		LT05_143029_20030529	38
2004	Landsat5_TM	LT05_143029_20040702	6
		LT05_143030_20040702	11
2005	Landsat5_TM	LT05_143029_20050619	0
		LT05_143030_20050619	19
2006	Landsat5_TM	LT05_143029_20060622	0
		LT05_143030_20060622	6
2007	Landsat5_TM	LT05_143029_20070625	0
		LT05_143030_20070625	11
2008	Landsat5_TM	LT05_143029_20080510	0
		LT05_143030_20080510	3
2009	Landsat5_TM	LT05_143029_20090614	5
		LT05_143030_20090716	12
2010	Landsat5_TM	LT05_143029_20100703	19
		LT05_143030_20100703	71
2011	Landsat5_TM	LT05_143029_20110604	0
		LT05_143030_20110604	2
2013	Landsat8_OLI	LC08_143029_20130609	0.05
		LC08_143030_20130609	6.91
2014	Landsat8_OLI	LC08_143029_20140527	0.34
		LC08_143030_20140527	8.39
2015	Landsat8_OLI	LC08_143029_20150717	2.29
		LC08_143030_20150717	7.91
2016	Landsat8_OLI	LC08_143029_20160516	6.99
		LC08_143030_20160516	3.52
2017	Landsat8_OLI	LC08_143029_20170620	0.8
		LC08_143030_20170620	8.97
2018	Landsat8_OLI	LC08_142029_20180522	0.03
		LC08_142030_20180522	2.35
2019	Landsat8_OLI	LC08_143029_20190525	0.35
		LC08_143030_20190525	4.04
2020	Landsat8_OLI	LC08_143029_20200527	0.14
		LC08_143030_20200527	2.66

Table S3. Values of NDVIveg and NDVIsoil.

Year	NDVIv	NDVIs
2000	0.797248	0.030334
2001	0.752873	0.008891
2002	0.813072	0.018738
2003	0.711846	0.026076
2004	0.718068	0.017333
2005	0.743112	0.027248
2006	0.786309	0.018308
2007	0.762101	0.026306
2008	0.587261	0.008304
2009	0.822923	0.027764
2010	0.702628	0.033905
2011	0.755456	0.008804
2013	0.836641	0.020191
2014	0.010129	0.010129
2015	0.848141	0.040466
2016	0.828082	0.020238
2017	0.858482	0.030346
2018	0.817579	0.020223
2019	0.816964	0.010122
2020	0.785177	*0.020173*
